# Patient Perception of Mobile Phone Apps for the Care and Prevention of Sexually Transmitted Diseases: Cross-Sectional Study

**DOI:** 10.2196/16517

**Published:** 2020-11-10

**Authors:** Lena Jakob, Theresa Steeb, Zeno Fiocco, Teodora Pumnea, Sophia Nomi Jakob, Anja Wessely, Christoph Clemens Rothenberger, Titus Josef Brinker, Lars Einar French, Carola Berking, Markus Vincent Heppt

**Affiliations:** 1 Department of Dermatology and Allergy University Hospital Munich Ludwig Maximilian University Munich Munich Germany; 2 Department of Dermatology Universitätsklinikum Erlangen Friedrich-Alexander-University Erlangen-Nürnberg Erlangen Germany; 3 Deutsches Zentrum für Immuntherapie Friedrich-Alexander-University Erlangen-Nürnberg Erlangen Germany; 4 Arbeitsstelle für Diagnostik und Evaluation Johann Wolfgang Goethe University Frankfurt Frankfurt Germany; 5 National Center for Tumor Diseases German Cancer Research Center Heidelberg Germany; 6 Department of Dermatology University Hospital Heidelberg Heidelberg Germany

**Keywords:** sexually transmitted diseases, sexually transmitted infection, mobile phone apps, health apps

## Abstract

**Background:**

In the emerging era of digitalization and electronic health, various health-related apps have been launched, including apps for sexually transmitted diseases. Until now, little has been known about how patients perceive the value of such apps.

**Objective:**

To investigate patient’s attitudes and awareness toward sexually transmitted disease–related apps in an outpatient sexually transmitted disease clinic setting.

**Methods:**

A cross-sectional study was conducted at a dermatovenereological outpatient unit between April and July 2019. Patients completed a self-administered questionnaire on their perceptions of the popularity and usefulness of sexually transmitted disease–related apps. Descriptive analysis was performed with expression of categorical variables as frequencies and percentages. For continuous variables, the median, range, and interquartile range were indicated. Contingency tables and chi-square tests were used to investigate associations between sociodemographic data and items of the questionnaire.

**Results:**

A total of 226 patients were surveyed (heterosexual: 137/193, 71.0%; homosexual: 44/193, 22.8%; bisexual: 12/193, 6.2%); 11.9% (27/225) had previously used health-related apps. Nearly half of the patients (97/214, 45.3%) specifically considered sexually transmitted disease–related apps useful, 47.8% (100/209) voted that they could supplement or support the consultation of a physician. Interestingly, only 35.1% (74/211) preferred a printed patient brochure on sexually transmitted diseases over downloading and using an app, but 64.0% (134/209) would download a sexually transmitted disease–related app recommended by their physician. General information regarding sexually transmitted diseases (93/167, 55.7%), evaluation of skin diseases based on photos or videos (78/167, 53.3%), information on the prevention of sexually transmitted diseases (76/167, 45.5%), mediation of nearby contact points or test sites (74/167, 44.3%), anonymous medical advice (69/167, 41.3%), and calculation of the risk of having a sexually transmitted disease (63/167, 37.3%) were rated as the most important features. Men were more likely than women to find sexually transmitted disease–related apps useful in general (*P*=.04; χ^2^=6.28) and to pay for such apps (*P*=.01; χ^2^=9.19). Patients aged <40 years would rather download an app recommended by their physician (*P*=.03; χ^2^=7.23), whereas patients aged >40 years preferred reading a patient brochure on sexually transmitted diseases (*P*=.02; χ^2^=8.14).

**Conclusions:**

This study demonstrated high general interest in the use of sexually transmitted disease–related apps in this sample of dermatovenereological outpatients. In particular, young age and male sex were significantly associated with a positive perception, underlining the high potential of apps in the prevention and early recognition of sexually transmitted diseases in this group. Future studies are warranted to validate these findings in other populations.

## Introduction

Mobile phones and tablets are being increasingly integrated into the daily lives of many people worldwide. Mobile health apps have a high potential to improve the quality and coverage of care; increase access to health information, services, and skills; as well as promote changes in health behaviors [1]. However, there is little regulatory control over the accuracy and medical validity of the information provided in apps [2]. Furthermore, privacy, moral, and ethical concerns have been raised [3,4]. Data extracted from digital health apps could be used to record, survey, monitor, influence, and discipline users [5].

Sexually transmitted diseases (STDs) represent a common but preventable cause for morbidity and serious medical complications. In our clinical experience, patients of STD clinics are usually young and especially prone to the use of modern technologies. Digital revolution did not only change their way of social interaction, in fact, in our clinical experience, it changed the risks associated with their sexual behavior, especially among men having sex with men. Meeting sex partners through geosocial networking apps is common among men having sex with men [6]. The use of internet- and app-based services, such as Grindr or Tinder, to facilitate sexual partnering is a known risk factor for the transmission of STDs and human immunodeficiency virus (HIV) [7-10].

In response, many online dating apps have established standard notification methods to inform users of recent HIV or STD exposure [7,11]. Other functions of health apps in the context of STDs aim to ensure adherence to antiretroviral therapy medication in patients who are HIV-positive by reminding them of antiretroviral therapy intake, providing health care via video consultations (mobile telemedicine), or through self-test kits (home tests) for detection of STDs [12,13]. Other common features of STD-related apps include sexual health promotion and educational information on prevention of STDs [14].

Given the strong emergence of various STD-related health apps, it is crucial to comprehend their implication in daily life as well as to document user experience and perception of those tools from a patients’ perspective. However, until now, little was known about how patients perceive the value of such apps. Hence, we aimed to examine how patients from an STD clinic perceive mobile health apps for STDs with a cross-sectional study.

## Methods

### Design and Procedure

A cross-sectional study including patients from the dermatovenereological outpatient clinic of the dermatology and allergy department of the university hospital of Munich was conducted between April and July 2019. This study was approved by the institutional review board of the university hospital (Ludwig Maximilian University Munich; approval number 19-336 KB). We closely adhered to the STROBE statement for cross-sectional studies for the reporting of this study [15,16].

### Setting, Participants, and Sampling

The dermatovenereological unit of the university hospital of Munich is a public outpatient clinic in downtown Munich, Germany. Munich represents a wealthy city with comparably low crime and drug use as well as high levels of education [17]. The unit mainly focuses on the diagnosis, treatment, and follow-up care of patients with sexually transmitted infections. Furthermore, patients with nonvenereal genital diseases such as candidiasis or chronic inflammatory dermatoses represent an important number of cases. These 2 patient groups are different in many ways. Patients presenting with STDs are most commonly young men having sex with men. In contrast, patients suffering from inflammatory diseases of the anogenital area are frequently postmenopausal women. During the period when the survey was conducted (April to July 2019), 969 individuals (1241 visits) presented to the unit, with a median age of 40.3 years, of which, 73.1% were male and 26.9% were female. The top 3 diagnoses using International Statistical Classification of Diseases, Tenth Revision (ICD-10) codes were HIV (B24.0; n=319), condyloma acuminata (n=190), and other STDs (A64.0; n=75). In that period, there were 104 patients with nonvenereal diseases that included multiple distinct diagnoses according to ICD-10 (L90.0, L30.9). All German-speaking adult patients (16 years or older) presenting to the unit were asked to complete a 3-page paper questionnaire by a physician (LJ, ZF, TP). Participation was voluntary, and all participants gave verbal informed consent in German before completing the questionnaire. The individual questions were not linked (ie, individual questions could be skipped to continue with the questionnaire). Refusals were not documented, and no incentives were provided. The study population was a convenience sample.

### Survey

As no validated survey tools for the objective of our study existed, the 3-page questionnaire was developed de novo based on a literature review and dermatovenereological expert consulting, and included questions on STD-related apps and basic demographic information (age; gender; place of residence—town, suburb, or rural area; and education).

In a multiple-choice question format, patients were asked about the reason for presenting to the unit on the day of the assessment, their sexual orientation, and their sexual risk behavior. Other studies [18] that exclusively focus on STDs in a high-risk population have used criteria such as the number of condomless anal intercourse encounters as risk criteria for acquiring an STD. By considering the number of patients with noninfectious diseases of the anogenital area, we estimated that the individual risk level for STDs for those in our study population was moderate. We asked participants if they had changed partners or had a sexual disease recently. Thus, the sexual risk behavior was operationalized by the number of sexual partners in the previous 6 months. This value was decided by our clinical experience in this specific population. Other questions addressed previous use of health-, and specifically, STD-related apps, perceptions of the relevance of those apps, as well as concerns regarding digital security. These questions were dichotomous; however, patients could also state that they were unsure. The full questionnaire is available in Multimedia Appendix 1. It was validated by 10 healthy volunteers (4 male, 6 female) for clarity and comprehensibility, and 5 physicians assessed the questionnaire for clinical validity referring to an external benchmark study [12] that was similar in content to our investigation. Based on their feedback, the questionnaire was revised to its final form. Completed questionnaires were sequentially numbered for data entry purposes but were not linked to any identifying patient information to assure irreversible anonymity.

### Data Analysis

Categorical variables were expressed as frequencies and percentages and were compared using 2-sided chi-square tests. *P*<.05 was considered statistically significant. For continuous variables, median, range, and interquartile range were reported. Statistical analyses were conducted with SPSS statistical software (version 25; IBM Corp).

## Results

### Characteristics of the Study Population

A total of 226 patients were included. Sociodemographic characteristics of the population are shown in Table 1.

The majority (75.2%, 170/226) presented with genital discomfort, which persisted for more than 6 weeks in 69.2% of patients (117/169). Additionally, most patients had already consulted a physician in person for their symptoms (124/162, 76.5%). Further reasons for presentation were examination for STDs (72/226, 31.9%), HIV treatment or consultation (20/226, 8.8%), skin cancer screening (16/226, 7.1%), or other (54/226, 23.9%).

The majority of patients had 0-2 different sex partners in the previous 6 months (126/176, 71.6%), and 28.4% (50/176) had more than 2 sex partners in the previous 6 months. Overall, 21.5% of patients had already been diagnosed with an STD prior to presentation (44/205). Regarding sexual orientation, patients were heterosexual (137/193, 71.0%), homosexual (44/193, 22.8%), or bisexual (12/193, 6.2%). Interestingly, 28.0% (58/207) had already made use of an app to arrange sexual encounters, underlining the moderate to high-risk sexual behavior of the study population (Table 1).

**Table 1:** Overview of the characteristics of the included patients in the cross-sectional study (N=226).

Characteristic		Value
**Sex (n=210** **), n (%)**		
	Female	49 (23.3)
	Male	161 (76.7)
**Age (in years; n=209)**		
	median (IQR)	37 (27-52)
	range	16-83
	mean (SD)	40.2 (15.2)
**Education (n=206), n (%)**		
	University degree	67 (32.5)
	General higher education entrance qualification	57 (27.7)
	Secondary school leaving certificate	47 (22.8)
	Lower secondary school leaving certificate	28 (13.6)
	Other degree	1 (0.5)
	No degree	6 (2.9)
**Residence (n=210), n (%)**		
	City or suburb	165 (78.6)
	Rural area	45 (21.4)
**Reason for appointment (n=226, multiple answers possible), n (%)**		
	Examination for STD	72 (31.9)
	Skin cancer screening	16 (7.1)
	HIV treatment or consultation	20 (8.8)
	Other reasons (eg, follow-up care)	54 (23.9)
	Genital discomfort	170 (75.2)
	Genital discomfort persisting for more than 6 weeks (n=169)	117 (69.2)
	Previous consultation of a physician due to genital discomfort (n=162)	124 (76.5)
**Previous diagnosis of an STD (≤6 months) (n=205), n (%)**		
	Yes	44 (21.5)
	No	161 (78.5)
**Sexual orientation (n=193), n (%)**		
	Heterosexual	137 (71.0)
	Homosexual	44 (22.8)
	Bisexual	12 (6.2)
**Number of sex partners (≤6 months) (n=176), n (%)**		
	0-2 sex partners	126 (71.6)
	>2 sex partners	50 (28.4)
**Previous use of an app to arrange sexual encounters (n=207), n (%)**		
	Yes	58 (28.0)
	No	149 (72.0)

### Previous Experience With Health Apps

In total, 34.1% (76/223) of the patients owned both a smartphone and a tablet, 59.6% (133/223) were owners of a smartphone only, and 2.2% (5/223) were owners of a tablet device only. Only 4.0% (9/223) had none of these devices. Most of patients (184/226, 81.4%) had already searched the internet prior to this survey in order to obtain health-related information, and additionally, 72.0% (152/211) had searched the internet before to acquire information about the specific complaints for which they were visiting the clinic.

When asked about previous experience with health-related apps, 11.9% (27/225) had previously made use of such apps, whereas the overwhelming majority (194/225, 86.7%) denied or were unsure (3/225, 1.3%). Apps that had already been used by the patients had been provided by health insurance (*DAK-Gesundheit*, *Allgemeine Ortskrankenkasse*, *Barmer Ersatzkasse*, *Techniker Krankenkasse*) or had been health-tracking apps offered by Apple, Android, Runtastic (fitness-tracking app), or Garmin.

Most patients (110/166, 66.3%) rated scientifically reliable information as the most important feature for health-related apps. For 52.4% (87/166), credibility of the provider as well as data security were important, followed by user convenience (86/166, 51.8%) and continuous availability (78/166, 47.0%); 41.0% (68/166) and 27.1% (45/166) considered a low price and an attractive layout, respectively, as critical.

### Attitude Toward STD-Related Apps

Nearly half of the patients (97/214, 45.3%) considered apps for patients with complaints in the intimate area or venereal diseases to be useful; 31.8% (68/214) and 22.9% (49/214) were unsure or thought that such apps were not useful for patients, respectively; and 47.8% (100/209) voted that STD-related apps could supplement or support the consultation of a physician, while 23.0% (48/209) were unsure. The vast majority did not believe that apps can replace the consultation by a physician (cannot be replaced: 184/209, 88.0%; unsure: 19/209, 9.1%). More than half of the patients (111/208, 53.4%) thought that apps can contribute to detecting diseases in the intimate area earlier. In addition, 46.9% of patients (98/209) were convinced that broad usage may reduce the spread of STDs (unsure: 67/209, 32.1%; cannot reduce the spread: 44/209, 21.1%). Furthermore, less than half of the patients did not agree with the statement that the broad use of such apps can save medical costs (disagree: 493/209, 4.5%; unsure: 61/209, 29.2%).

**Figure 1:**
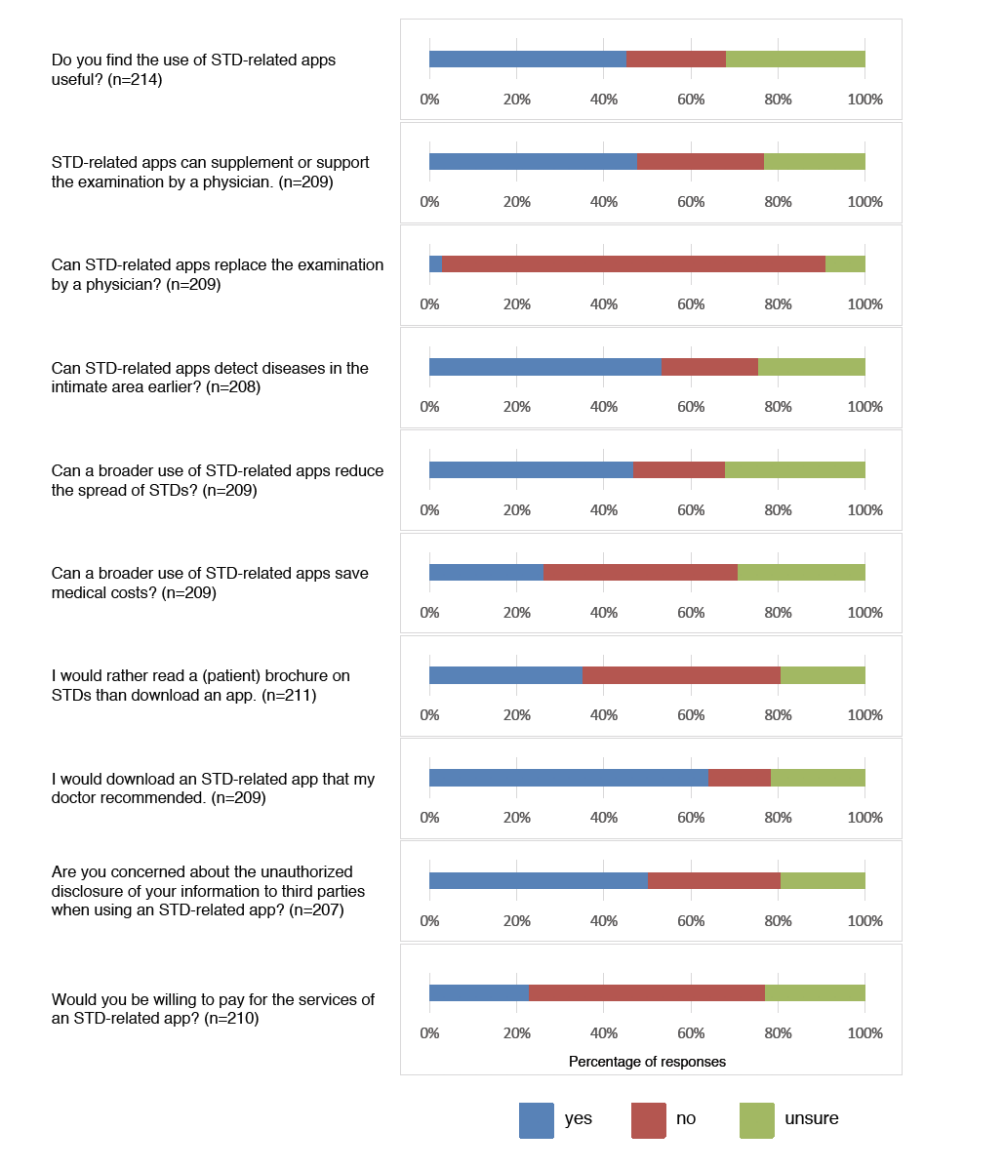
Patients' answers regarding their attitudes toward STD apps.

Interestingly, only 35.1% of patients (74/211) preferred a printed patient brochure on STDs over downloading and using an app. Half of the patients (104/207, 50.2%) had concerns about the unauthorized disclosure of information to third parties, while 30.4% (63/207) did not have concerns and 19.3% (40/207) were unsure. However, 64.0% of the patients (134/209) would download an STD-related app recommended by their physician, and only 22.9% (48/210) were willing to pay for services provided by an STD-related app (Figure 1).

We also asked the patients which app features they considered important for patients with complaints in the intimate area. The majority reported general information regarding STDs to be important (93/167, 55.7%), followed by the evaluation of skin diseases based on photos or videos (78/167, 53.3%), information on the prevention of STDs (45.5%76/167), mediation of nearby contact points or test sites (74/167, 44.3%), anonymous medical advice (69/167, 41.3%), and calculation of the risk of having an STD (63/167, 37.3%). Less than one-third of the patients found treatment plans with reminders for medication (46/167, 26.9%), mailing of test kits to be used at home (43/167, 25.7%), and verification of STD status of sexual partners or safe dating (24/167, 14.4%) to be relevant.

### Association Between Sociodemographic Data And Attitude Toward STD-Related Apps

#### Sex (Men Versus Women)

In comparison to women, men were more likely to find STD-related apps useful in general (*P=*.04; χ^2^=6.28). However, in terms of possible features, men were less frequently interested than women in apps on information about prevention of STDs (*P=*.02; χ^2^=5.82). In our sample, more men than women agreed that STD-related apps can detect STDs earlier (*P*<.001; χ^2^=15.72) and that medical costs can be saved (*P=*.006; χ^2^=10.37). In addition, more men than women preferred reading a brochure on STDs over downloading an app (*P=*.006; χ^2^=10.18). There was also an association between the willingness to pay for the services provided by an app and male sex (*P=*.01; χ^2^=9.19)—men were more willing to pay than women were (Table 2).

**Table 2:** Association between sociodemographic data and attitude toward STD-related apps.

Item of the questionnaire	*P* value			
	Sex (male vs female)	Residence (city/suburb vs rural)	Age (<40 years vs ≥40 years)	Sexual risk behavior (0-2 vs >2 partners)
Generally useful?	.04	.08	.08	.04
Supplement or supportive?	.51	.13	.25	.63
Replace examination?	.45	.55	.27	.09
Earlier detection?	.001	.027	.001	.02
Reduce spread of STDs?	.07	.13	.11	.02
Save medical costs?	.006	.03	.009	.06
Prefer brochure?	.006	.01	.02	.16
Download recommended app?	.16	.48	.027	.72
Disclosure of personalized data?	.24	.44	.12	.12
Willing to pay?	.01	.04	.052	.29

#### Residence (City/Suburb Versus Rural Area)

Patients living in a city or suburb versus those living in rural areas agreed more frequently to the statements—STD-related apps can detect diseases in the intimate area earlier (*P=*.03; χ^2^=7.25) and apps can contribute to saving medical costs (*P=*.03; χ^2^=6.75). Furthermore, patients living in a city or suburb, compared to participants from rural areas, were more willing to pay for the services offered in such apps (*P=*.04; χ^2^=6.73).

#### Age (<40 Years Versus ≥40 Years)

Patients <40 years of age considered an attractive layout (*P=*.04; χ^2^=4.25), evaluation of skin diseases based on photos or videos (*P=*.004; χ^2^=8.498), and mailing of test kits for home usage (*P=*.002; χ^2^=9.77) as important features for STD apps more frequently than those >40 years of age. Additionally, patients <40 years were more likely to agree that diseases may be detected in a timely manner (*P*< 0.001; χ^2^=18.82) and that medical costs can be saved through the usage of STD-related apps (*P=*.009; χ^2^=9.40) than those >40 years of age. Furthermore, patients <40 years of age would rather download an app recommended by their physician instead of reading a printed brochure (*P=*.03; χ^2^=7.23), whereas patients aged >40 years preferred reading a patient brochure on STDs instead of downloading an app (*P=*.02; χ^2^=8.14).

#### Sexual Risk Behavior (0-2 Partners Versus >2 Partners in the Previous 6 Months)

Regarding preferred features of STD-related apps, evaluation of skin diseases based on photos or videos was chosen more frequently by patients with 0-2 sexual partners in the previous 6 months than it was chosen by those with >2 partners (*P=*.01; χ^2^=6.19). In addition, patients with 0-2 sexual partners in the previous 6 months were more interested in home test kit mailing for STD self-tests (*P=*.04; χ^2^=4.38) than those with >2 partners in the previous 6 months were. Patients with >2 partners were more interested in safe sex partner dating or verification of the STD status of the partner than those with 0-2 partners were (*P=*.003; χ^2^=8.86).

#### Other Factors

Patients without an STD in the previous 6 months indicated more frequently that self-test kits for STDs (*P=*.005; χ^2^=8.05) and individual STD risk assessment (*P=*.008; χ^2^=7.05) were important features of STD apps compared to those with a recent STD. Patients who did not use apps to arrange sexual encounters chose the feature evaluation of skin diseases based on photos or videos (*P=*.001; χ^2^=11.797) more frequently than those regularly using apps did. Mailing of test kits (*P*<.001; χ^2^=20.64) was found to be crucial for those who had already made use of dating apps in the past than for those who had not used any apps.

## Discussion

In recent years, scientists have evaluated many apps in the field of sexual health [14,19]. We believe it is necessary to ask the target groups of STD-related apps about all aspects of those digital services directly, to explore the patient perspective. The overall goal of this study was to explore the patient perspective of STD-related apps, and thus, to guide the further development of apps based on these identified patient-centered preferences.

We interviewed 226 patients, who presented to an outpatient STD clinic for various reasons, about their perception of STD-related apps. The study population was heterogeneous and included both women and men, with men being predominant. In our clinical experience, the proportion of men having sex with men was low compared to those of other STD clinics. This may be explained by the fact that this STD clinic belongs to the department of dermatology where many patients are treated with chronic inflammatory conditions or other primary nonvenereal diseases of the genital area. Nevertheless, 28.4% of the patients (126/176) reported more than 2 sexual partners in the previous 6 months, and 28.0% (58/207) indicated that they had already used an app at least once to make sexual contacts. From these data, we conclude that the sexual behavior of the sample presented in this study is most likely to correspond to moderate to high risk for STDs.

To our knowledge, this is the first study to include an analog survey of patients seeking medical advice from a physician on their perception of the many aspects of digital health services regarding sexual health. In contrast to the focus of other studies [20,21], we did not focus exclusively on specific high-risk groups such as men having sex with men, individuals who are HIV-positive, or users of geosocial sexual networking apps but interviewed the patients regardless of their sexual orientation, HIV status, or internet expertise to gain a broad opinion of STD-related apps. We believe that this approach is necessary for 2 reasons. First, many existing STD apps are aimed at a broad usership. Second, in our experience, patients with genital discomfort usually do not know for themselves whether their complaints are due to an STD or some other nonsexually transmitted disease. In this study, we intended to encompass the perspective of these distinct patient groups.

Despite the fact that most of the patients surveyed in this study had no prior experience with health apps, the majority used the internet to get health information in general: 72% of the study population (152/211) affirmed that they had searched the internet for information about the specific complaint that they presented at the STD clinic and, additionally, 64.0% (134/209) indicated that they would download an STD app recommended by their physician. These findings demonstrate a great general interest of STD patients in acquiring digital health information before and after visiting a doctor. It also indicates that even if the vast majority did not believe that apps could replace a consultation by a physician, they would generally appreciate additional information.

There might be a group of patients with similar complaints who had looked up information on the internet, was satisfied, and thus never presented to the clinic, and therefore, was not represented in the study. However, surveys of physicians who offered app-based care for STD patients showed referral rates to clinics of almost 100% [12]. This and other studies [14,19] that evaluated a range of STD-related apps support the statements of our study population that mobile medical apps in the field of STDs do not replace visits to clinics and form an argument against the existence of this second unrepresented group.

Interestingly, despite the low experience of our study population with health apps, the majority preferred to obtain health information delivered via app over reading a patient brochure in contrast to the findings of other studies [22]. Thus, the results highlight the great potential for digital health app providers in targeting multiple patient groups with complaints in the genital area.

When questioned about important aspects of digital health apps, the most valued aspects were scientifically reliable information, followed by data security and a trustworthy provider. These items highlight the strong need for reliability and confidentiality. Of all functions, general information and the evaluation of skin diseases based on photos or videos were the features most frequently desired. We estimate that these features could be useful for both patients with STDs and patients with nonvenereal genital complaints. In contrast, functions to find partners for safe sex, home-based tests, and treatment plans with reminders of medication intake (eg, for antiretroviral therapy) were chosen less frequently. This could be due to the fact that high-risk groups such as men having sex with men or patients who are HIV-positive were relatively underrepresented in this population [20]. In particular, the choice of apps for safe sex partner dating and verification of the STD status of the partner was very significantly associated with the number of sexual contacts within the previous 6 months, highlighting that individual sexual behavior is closely related to desired target functions of the apps. In contrast, patients with fewer sexual partners within the previous 6 months were more interested in an evaluation and recognition of genital diseases, and especially, in home-based tests. These results underline the great interest of patients in an anonymous diagnosis or consultation without the necessity of a direct contact with a physician.

We are aware that this study has several limitations. The sample comprised 226 patients presenting to the venereological outpatient unit. First, this sample size is relatively small, and second, it was not sampled in a random fashion but depending on the availability of patients. The questionnaire has not been validated and was developed de novo. Thus, the questionnaire may lack validity and the results presented here may not be fully generalizable to other populations or other STD clinics and are at risk of sampling bias. Although the survey was anonymized, patients were likely known to the treating physicians, which may have altered the response in either direction, potentially biasing the results. The items of the questionnaire focused primarily on what patients might do outside of a clinic and did not include clinic-specific aspects such as communication with staff. Furthermore, this sample was a selection of patients physically presenting to the unit. Thus, the value and evaluation of STD-related apps among patients who did not present to seek STD consultation and treatment remains unclear. The response rate on the individual questions varied but was generally high. The question with the lowest response rate (77.9%) was the one about the number of sexual partners. This is consistent with other studies that investigated sexual risk behavior [20]. It could be due to the fact that participants perceive answering intimate questions as uncomfortable or, despite being assured anonymity, they are not sure about the confidential treatment of their data [18].

In our study population, men were generally more open to the use of STD-related apps than female patients. In comparison to female participants, men rated STD-related apps as more useful, cost-effective, and more reliable in early detection of STDs. Furthermore, they were more likely than females were to be willing to spend money for the services of an STD-related app. This may be explained by the gender disparity of our study population in terms of distribution of STDs and nonvenereal genital diseases. In our STD department, we observe predominantly male patients with STDs. Furthermore, we tend to have more female patients presenting with nonvenereal genital inflammatory diseases. This perception is consistent with epidemiologic data on the sex distribution of STDs in Germany [23]. Gender disparity in the distribution of STDs could be a reason for the higher interest of male participants in STD-related apps in general, but could it possibly explain why, in this study, men showed significantly less interest in apps with information on prevention of STDs than women? Higher sexual risk behavior might be associated with a lack of interest in prevention of STDs. We believe these data underline the hypothesis that women and men as well as specific risk groups should be addressed individually with tailored information in order to achieve a higher engagement in adopting preventive behavior. Further studies are needed in order to improve future STD prevention and information efforts and to validate our findings in other populations.

## Appendix

Multimedia Appendix 1 Original questionnaire.

## Abbreviations

HIV: human immunodeficiency virus
ICD-10: International Statistical Classification of Diseases, Tenth Revision
STD: sexually transmitted disease
STROBE: Strengthening the Reporting of Observational Studies in Epidemiology
