# Supplementary material for: Patient Perception of Mobile Phone Apps for the Care and Prevention of Sexually Transmitted Diseases: Cross-Sectional Study
Source: JMIR Mhealth Uhealth. 2020 Nov 10;8(11):e16517. doi: 10.2196/16517 (PMC7685917; doi:10.2196/16517)
Supplement: Multimedia Appendix 1 [file mhealth_v8i11e16517_app1.pdf]

What is the reason for your presentation to the dermatology clinic today?

☐ Examination for sexually transmitted diseases

☐ Complaints in the genital area :

If you have genital problems:
Do they last longer than 6 weeks?

☐ Yes ☐ No ☐ Don’t know

Have you ever seen a doctor for these complaints?

☐ Yes ☐ No ☐ Don’t know

☐ Skin cancer screening

☐ Treatment/counceling for HIV

☐ Other: ______________________________

Do you own one of the following devices? (multiple answers possible)

☐ Smartphone
☐ Tablet (e.g. „iPad“)
☐ None

Do you use the Internet to get information about your health?

☐ Yes ☐ No ☐ Don’t know

If so, have you searched the Internet for the cause of your complaints?

☐ Yes ☐ No ☐ Don’t know

Have you ever used health-related apps?

☐ Yes ☐ No ☐ Don’t know

If so, which ones?

____

What do you find most important in health-related apps? (multiple answers possible)

☐ Attractive design ("layout")
☐ Scientifically substantiated information
☐ Trusted providers
☐ Free of charge / low price
☐ User-friendly
☐ Security of information/data security/ anonymity
☐ 24/7 availability

☐ Other : ______________________________

Do you find the use of apps for patients with genital dyscomfort / sexual transmitted diseases ("STD apps") useful?

☐ Yes ☐ No ☐ Don’t know

Which functions do you find important in an STD app? (multiple answers possible)

- ☐ Anonymous medical advice without personal contact
- ☐ Evaluation of skin diseases based on photos or videos
- ☐ Home test kits for self testing
- ☐ Determination of personal risk for STD's
- ☐ Information on the prevention of venereal diseases
- ☐ General information on STD's
- ☐ Treatment plan with reminder to take HIV medication (ART)
- ☐ Information on nearest STD clinic/test points nearby
- ☐ "Safe - Sex Dating Service" / Verification STD status of sexual partners

Do you think that STD apps can <u>complement</u> or <u>support</u> the examination by a physician?

☐ Yes ☐ No ☐ Don't know

Do you think STD apps can <u>replace</u> a visit to the doctor?

☐ Yes ☐ No ☐ Don't know

Do you believe that STD apps can help to detect genital diseases earlier?

☐ Yes ☐ No ☐ Don't know

Do you believe that a wider use of STD apps can reduce the spread of STD's?

☐ Yes ☐ No ☐ Don't know

Do you believe that a wider use of STD apps can save medical costs?

☐ Yes ☐ No ☐ Don't know

I would rather read a (patient) brochure on STD's than download an app.

☐ Yes ☐ No ☐ Don't know

I would download an STD app that my doctor recommended.

☐ Yes ☐ No ☐ Don't know

Do you have concerns about unauthorized disclosure of your information to third parties when using an intimate app?

☐ Yes ☐ No ☐ Don't know

Currently, costs for the use of intimate apps are not covered by public health insurance companies.

Would you be willing to pay for the services of an STD app?

☐ Yes ☐ No ☐ Don't know

If so, how much would you be willing to spend on these services?

☐ <20€ ☐ 20-50€ ☐ >50 €

Personal information:

Are you male or female?

☐ Male ☐ Female

How old are you?:

☐_______ years old

Where do you live?

☐ City (e.g. Munich city and surrounding area, other city)
☐ Rural area

Which is your highest level of education?

☐ No school qualification
☐ Secondary school
☐ Higher education entrance qualification
☐ Lower secondary school
☐ Other degree (indicate):
☐ University degree

How would you describe your sexual orientation?

☐ Heterosexual
☐ Homosexual
☐ Bisexual

Which statement about your sexual contacts is true?

☐ > 2 different sexual partners in the past 6 months
☐ 0 - 2 different sexual partners in the past 6 months

Have you been diagnosed with an STD (e.g. herpes, syphilis or similar) in the last 6 months?

☐ Yes
☐ No

Have you ever used an app to make sexual contacts?

☐ Yes
☐ No

You have now reached the end of the questionnaire. Thank you very much for your participation.
